# Uterus transplantation; first data on neurologic, neuropsychiatric, and physical examination follow-up of children up to 6 years of age

**DOI:** 10.1093/humrep/deaf178

**Published:** 2025-09-13

**Authors:** E Wentz, B Hagberg, H Hagberg, H Bokström, M Brännström

**Affiliations:** Department of Psychiatry and Neurochemistry, Institute of Neuroscience and Physiology, University of Gothenburg, Gothenburg, Sweden; Region Västra Götaland, Department of Neuropsychiatry, Sahlgrenska University Hospital, Gothenburg, Sweden; Gillberg Neuropsychiatry Centre, Institute of Neuroscience and Physiology, University of Gothenburg, Gothenburg, Sweden; Department of Obstetrics and Gynecology, Institute of Clinical Sciences, University of Gothenburg, Gothenburg, Sweden; Department of Obstetrics and Gynecology, Institute of Clinical Sciences, University of Gothenburg, Gothenburg, Sweden; Department of Obstetrics and Gynecology, Institute of Clinical Sciences, University of Gothenburg, Gothenburg, Sweden; Stockholm IVF-EUGIN, Stockholm, Sweden

**Keywords:** uterus transplantation, offspring, long-term follow-up, cognition, neurodevelopment

## Abstract

**STUDY QUESTION:**

What are the outcomes regarding cognition, development, physical, and psychiatric status of children born after uterus transplantation (UTx) up to 6 years after birth?

**SUMMARY ANSWER:**

The long-term and very long-term outcomes of children born after UTx indicate normal cognitive, neuropsychiatric, and physical development.

**WHAT IS KNOWN ALREADY:**

Previous cohort studies of children born after UTx and followed up to 2–3 years indicate normal neurodevelopment and occasional cases of minor malformations.

**STUDY DESIGN, SIZE, DURATION:**

A prospective cohort study was performed on 11 offspring after UTx. All children (7 boys, 4 girls) were examined at age 2.5 years and eight children (5 boys, 3 girls) were examined at age 6 years.

**PARTICIPANTS/MATERIALS, SETTING, METHODS:**

The cognitive evaluations, Bayley-III test and WPPSI-IV, were performed at age 2.5 years (n = 11) and age 6 years (n = 8), respectively. Parental questionnaires pertaining to neurodevelopmental and behavioural problems, including the Strengths and Difficulties questionnaire (SDQ) and the ESSENCE-Q, were administered. All children (age 2.5 years: n = 11; age: 6 years: n = 8) underwent physical examinations.

**MAIN RESULTS AND THE ROLE OF CHANCE:**

Cognitive skills showed results within the normal range at both age 2.5 years and age 6 years. According to the SDQ, emotional problems were the most common symptoms, affecting two children at age 2.5 years and two children at age 6 years. Three children scored above the cut off on the ESSENCE-Q at age 2.5 years, and one child continued to score ‘high’ at age 6 years. At the first examination, three children had asthma, and speech problems were observed in five children. Among those who were also assessed at age 6 years, these problems had abated. At age 6 years, one child was considered hyperactive, and another child exhibited vocal tics. Developmental and behavioural deviations were observed almost exclusively in the boys.

**LIMITATIONS, REASONS FOR CAUTION:**

Limitations of the study include the small sample size, and the lack of a comparison group. The small sample does not offer enough statistical power, and no firm conclusions can therefore be drawn based on the reported deviances.

**WIDER IMPLICATIONS OF THE FINDINGS:**

The long-term and very long-term outcomes of children born after UTx indicated normal cognitive development. A minority had minor physical and developmental problems, including asthma and speech problems at age 2.5 years, but most of these symptoms subsided by age 6 years. Boys seemed to be over-represented regarding developmental and behavioural deviations. The small sample size limits the ability to generalize the findings to all children born after UTx.

**STUDY FUNDING/COMPETING INTEREST(S):**

The study was supported by The Swedish research council (Grant/Award Numbers: Dnr 2023-02035 (H.H.), 2024-03487 (M.B.)), the Swedish state under the agreement between the Swedish Government and the country councils, the ALF-agreement, grant/Award-Numbers: ALFGBG-1005108 (H.H.), ALFGBG-965535 (M.B.), Jane and Dan Olsson Foundation for Science (2020-09 (M.B.)), and Knut and Alice Wallenberg Foundation (2017.0363 (M.B.)). There are no conflicts of interest for any of the authors.

**TRIAL REGISTRATION NUMBER:**

NCT01844362, NCT02987023.

## Introduction

Uterus transplantation (UTx) is a novel and groundbreaking fertility treatment designed for women with absolute uterine factor infertility, a condition caused by congenital absence of the uterus, surgical removal, or the presence of a non-functional uterus incapable of sustaining pregnancy. Unlike other infertility treatments, UTx is unique in that it enables pregnancy and childbirth through organ transplantation, offering a reproductive solution where none previously existed. The world’s first live birth following UTx occurred in Sweden in 2014 (Brannstrom *et al.*, 2015). Since then, more than 100 UTx procedures have been performed worldwide (Brannstrom *et al.*, 2023), resulting in over 40 documented live births (Brannstrom *et al.*, 2025).

Despite the remarkable progress in UTx, the long-term health outcomes of children born through this procedure remain unexplored. Pregnancy following UTx requires immunosuppression (IS) to prevent organ rejection, similar to other solid organ transplantations (SOTs). IS typically involves calcineurin inhibitors, such as tacrolimus, corticosteroids, and the antiproliferative agent azathioprine (AZA), temporarily to reverse rejection episodes, or continuously during pregnancy. Corticosteroids and AZA present a lower risk to the foetus (Chandra *et al.*, 2019). Studies on pregnancies after SOTs have indicated an association with increased risks of complications such as preterm birth, foetal growth restriction, and infant mortality (Kallen *et al.*, 2005). Research on children born to SOTs recipients has identified an increased prevalence of recurrent infections, asthma, cerebral palsy, developmental delays, and congenital malformations (Willis *et al.*, 2000). Additionally, the use of IVF, which is a necessity following UTx, increases the risk of perinatal complications such as low gestational age, reduced birth weight, and increased preterm birth rate after UTx, as shown after IVF treatment (Chen *et al.*, 2024). Moreover, UTx pregnancies present unique physiological challenges, since the uterus often comes from post-menopausal donors, which may result in an altered microvascular environment. Additionally, the vascular supply to the transplanted uterus differs from a naturally developed uterus, relying exclusively on bilateral uterine arteries rather than additional ovarian and vaginal arteries in the physiological situation. Collectively, all these factors raise questions about potential impacts on foetal development and neonatal health of children born from a transplanted uterus.

While early studies on children born to UTx recipients have indicated promising outcomes, comprehensive long-term data remain limited. The first cohort of UTx children, born from the initial Swedish clinical trial, was followed up to 2 years, revealing no major malformations and only one case of inguinal hernia (Brannstrom *et al.*, 2022). In a study from the USA of UTx children followed up to 2 years, delayed expressive communication skills were seen in 1 out of 14 children and minor congenital malformations (urethral displacement in female infant, cryptorchidism in male infant) were seen in two children (Schulz *et al.*, 2022). In a case report of two UTx children from Czech Republic, the two children were followed with neurodevelopmental tests at age 2 and 3 years of age, and the neurodevelopmental scores were found to be within the normal ranges (Janota *et al.*, 2023).

At Sahlgrenska University Hospital, we have followed a cohort of 11 consecutive children born after UTx, including the world’s first UTx-born child from 2014 (Brannstrom *et al.*, 2015). The objective of this study is to assess the children’s physical, cognitive, and psychiatric development at 2.5 and 6 years of age. We hypothesized that most children would exhibit a normal cognitive profile and overall healthy physical and mental status.

## Materials and methods

### Ethical approval

The children are from two UTx studies with approvals from the Regional Ethics Committee, University of Gothenburg, Sweden (Nos 88-12 and 362-16). The initial study ‘Research study for clinical application of UTx and embryo transfer to achieve live birth’, in 2012–2013, included nine live donor UTx procedures with laparotomy techniques at donor hysterectomy and at transplantation. The second study ‘Uterus transplantation with robotic-assisted uterus retrieval’, in 2017–2019, included eight live donor UTx procedures with robotic-assisted laparoscopy at donor hysterectomy and laparotomy at transplantation.

Written informed consent was obtained from the recipients, their male partners, and live donors.

### Participants

This study included 11 children (4 girls, 7 boys), all recruited consecutively after being born by a mother with a history of UTx. They constitute the first 11 children in a case series born after UTx in Sweden including the first child born after UTx in the world. The children were born between 2014 and 2020.

The eight mothers of the 11 children (siblings from three mothers were included) were included in either of the two studies: ‘Research study for clinical application of UTx and embryo transfer to achieve live birth’, with surgeries by laparotomy in both live donors and recipients in 2012–2013; and ‘Uterus transplantation with robotic-assisted uterus retrieval’, with robotic-assisted live donor hysterectomy and laparotomy in recipients in 2017–2019. Details on characteristics of surgeries, recipients, and source of graft (live donor) are provided elsewhere (Brannstrom *et al.*, 2020a, b, 2014). In summary, all mothers had absolute uterine factor infertility due to uterine absence, with seven mothers having the Mayer–Rokitansky–Küster–Hauser syndrome with congenital uterine absence and one mother having undergone radical hysterectomy for cervical cancer (mother 1, Table 1). The mothers were otherwise healthy, non-smokers, and with BMI between 18.0 and 26.0 kg/m^2^. All pregnancies were achieved by IVF, from ovarian stimulation cycles conducted either before UTx (child 1–4, 9, 11) or after UTx (child 5–8, 10). The pregnancies were followed in a standardized way from gestational week 10, with regular clinical visits and laboratory tests every second week until gestational week 34 and then weekly, as described in more detail elsewhere (Brannstrom *et al.*, 2022).

**Table 1.** Perinatal and anthropometric data at age 2.5 and 6 years.

Code	Mothers’ codes of laparotomy (1–6) and of robotic (#1–#2) UTx trials;^(y,z)^	Sex	Born gestational week	Birth weight (g) (Deviation from expected)	Birth length (cm)	Birth head circumference (cm)	Breast-feeding (months)	Age at first developmental assessment, T1 (years)	Weight at first assessment, T1 (kg) (SD**)	Height at first assessment, T1 (cm) (SD**)	Head circumference, T1 (cm) (SD)	BMI at first assessment, T1 (SD**)	Age at second developmental assessment, T2 (years)	Weight at second assessment, T2 (kg) (SD**)	Height at second assessment, T2 (cm) (SD**)	BMI at second assessment, T2 (SD**)
1	1^y^	M	31 + 6	1,775 (−9%)	40	28.5	0	2.33*	17 (+1.7)	94 (+0.6)	51.5 (+0.6)	19.24 (+1.6)	6.25	27.8 (+1.7)	127.2 (+1.4)	17.2 (+1,25)
2	3^y^	M	35 + 0	2,700 (+4%)	46	35.5	0	2.5*	14.3 (+0)	NAa	54 (+2.6)	NAa	6.20	18.3 (−1.5)	112 (−1.2)	14.4 (−1.1)
3	2^y^	M	34 + 4	2,335 (−7%)	44	33.0	3	2.65*	14.2 (−0.2)	91 (+0.3)	51 (+0.1)	17.15 (+0.3)	6.47	22.4 (−0.1)	119 (−0.4)	15.8 (+0)
4	4^y^	M	34 + 4	3,074 (+23%)	47	35.0	17	2.68*	15.4 (+0.3)	89 (−1.3)	No information	19.4 (+1.8)	6,63	25.4 (+0.7)	117 (−1.0)	18.6 (+1.8)
5	5^y^	F	35 + 3	2,552 (−4%)	46	33.5	4	2.5*	12.3 (−1.0)	93 (+0.4)	Could not be assessed	14.2 (−2.0)	6, 34	21.6 (−0.2)	119 (−0.2)	15.3 (−0.2)
6	3^y^	F	37 + 0	2,600 (−13%)	44	34.0	0	2.71	12.2 (−1.2)	86 (−2.0)	No information	16.5 (+0)	6.16	17.4 (−2.0)	109.3 (−2.0)	14.56 (−0.7)
7	6^y^	F	37 + 1	2,376 (−12%)	45	34.0	8	2.38	16.2 (+1.5)	91 (+0)	No information	19.6 (+2.0)	6.21	23.8 (+0.7)	117.5 (−0.3)	17.24 (+1.2)
8	5^y^	M	35 + 6	2,745 (−1%)	48	34.5	4	2.67*	13.6 (−0.7)	93.5 (−0.4)	No information	15.6 (−1.0)	6.16	20.1 (−0.8)	117.2 (−0.25)	14.6 (−1.0)
9	#2^z^	M	36 + 1	2,894 (3%)	48	34.0	6	3.0*	16.2 (+0.5)	99.5 (+0.8)	No information	16.4 (−0.1)	NA	NA	NA	NA
10	6^y^	F	38 + 0	3,078 (−4%)	47	35.0	12	2.82	15.2 (+0.5)	92 (−0.3)	48.5 (−0.9)	18 (+1.0)	NA	NA	NA	NA
11	#1^z^	M	37 + 2	3,390 (11%)	49	37.0	4	2.57	15.6 (+0.7)	93.3 (+0)	50 (−0.5)	17.9 (+1.0)	NA	NA	NA	NA

The children with codes 2 and 6, 5 and 8, as well as 7 and 10 are siblings (not twins). Weight, height, and head circumference have been adjusted for gestational age at birth.

* Age corrected to gestational age.

** SD is the standard deviation from the mean value based on Swedish population-based reference charts (Karlberg and Luo, 2000); NA, not applicable; NA^a^, was not possible to measure; M, male; F, female.

y Brannstrom *et al.* (2022).

z Brannstrom *et al.* (2020a).

The maintenance IS during the pregnancies was identical in all women (Ekberg *et al.*, 2023) and consisted of tacrolimus (median (range) through levels of 6.1 (5.6–6.8) mg/ml), azathioprine (1 mg/kg daily), and prednisolone (5 mg daily). During pregnancies of two children (children 1 and 7), the mothers experienced one episode each of mild rejection and received additional treatment with corticosteroids (intravenous methylprednisolone 500 mg daily for 3 days and then 20 mg oral prednisolone, tapered to 5 mg daily over a 5-week period).

Breastfeeding did not take place for three children, two of whom had the same mother. Table 1 shows data on breastfeeding, including duration.

All children were assessed at 2.5 years of age, and eight children were assessed at 6 years of age. There were no dropouts, but the data collection ended in September 2023 as the three youngest would not turn 6 until 2025/2026. All children were born by Caesarean section. Seven children were born preterm (31 + 6 weeks (N = 1), 34–35 weeks (N = 5), 36 weeks (N = 1)); four deliveries were acute (preeclampsia (N = 2), intrahepatic cholestasis in pregnancy (ICP) (N = 1), preterm premature rupture of membranes, ICP, and preeclampsia (N = 1)), and three preterm deliveries were elective (35+ weeks was a compromise between foetal maturity and risk for major obstetrical complications during the last weeks of gestation in the early stages of the initial study). One child was born at 38 gestational weeks (Table 1).

### Instruments for age 2.5 years (T1)

#### Cognitive assessment

Bayley Scales of Infant and Toddler Development, third edition (Bayley-III) assesses the developmental functioning of infants/toddlers between 1 and 42 months of age, to identify children with developmental delay. Five domains are assessed: Cognitive, Language, Motor, Social-Emotional, and Adaptive. The two latter domains are conducted using a primary caregiver questionnaire and were not used in the present study. The Cognitive, Language and Motor assessments are conducted using items administered to the child, and there are a series of play tasks for assessing a child’s level of functioning across these three domains (Bayley, 2006). Compared to the WPPSI-IV (see below: ‘Instruments for age 6 years (T2): Cognitive assessment’), the Bayley test is more reliable in testing the youngest children because the scale is from 0 years. In the study group, some children were born preterm and needed to be adjusted to 2.5 years.

#### Questionnaires

The Strengths and Difficulties Questionnaire (SDQ) 2–4 years, parental version, was used (Croft *et al.*, 2015). SDQ 2–4 is an extensively used questionnaire to assess child psychopathology between ages 2 and 4 years. The form consists of five subscales: Emotional, Conduct, Hyperactivity, Peer problems, and Prosocial, with five items per subscale. An SDQ total score is calculated encompassing four subscales, excluding the Prosocial subscale. The answers are constructed as a three-point Likert scale rated between 0 and 2 (0 = not true; 1 = somewhat true; 2 = certainly true). The total score can vary between 0 and 40 points, and a cut-off score of >10 has been suggested in Sweden for 3-year-olds (Dahlberg *et al.*, 2020). The questionnaire has shown valid psychometric properties (Croft *et al.*, 2015).

ESSENCE-Q-REV is a parental interview consisting of 12 questions screening for symptoms of ESSENCE (Early Symptomatic Syndromes Eliciting Neuropsychiatric Clinical Evaluation) (Gillberg, 2010). The interview covers the child’s developmental problems, before the age of 6 years, including symptoms of ADHD, autism spectrum disorder (ASD), feeding and eating, speech and language, temper tantrums, and epilepsy. The parent can choose between ‘yes’, ‘no’, or ‘maybe’. The instrument has shown good psychometric properties as a ‘first-step screening tool’ (Hatakenaka *et al.*, 2016).

Modified Checklist for Autism in Toddlers (M-CHAT) (Robins *et al.*, 2001) is a parental screening instrument for ASD between age 16–30 months. It encompasses 20 questions that can be answered with either ‘yes’ or ‘no’. A total score of 0–2 points is considered as ‘Low risk’; 3–7 points as ‘Medium risk’, 8–20 points as ‘High risk’ of ASD.

### Instruments for age 6 years (T2)

#### Cognitive assessment

The Wechsler Preschool and Primary Scale of Intelligence—Fourth Edition (WPPSI-IV) was used. It measures the cognitive ability of children 2 years 6 months to 7 years 7 months. For ages 4:0–7:7, the test framework is organized into five Primary Index Scales: Verbal Comprehension, Visual Spatial, Fluid reasoning, Working Memory, and Processing Speed. The Full Scale includes all subtests in each scale at the Primary Index Scale level (Wechsler, 2012).

#### Questionnaires

SDQ 4–16 years, the parental version, encompasses the same subscales as the 2-4 version. According to the Swedish norms, the total scale’s normal range is 0–10 (Malmberg *et al.*, 2003).

ESSENCE-Q-REV is described above in the section for age 2.5 years.

Five-to-fifteen (FTF) is a parental questionnaire for children between age 5 and 15 years focusing on ADHD, but also other psychiatric symptoms coexisting with ADHD. The form includes 181 items. Scoring above the 90th percentile in a domain was classified as screening positive. The instrument is valid and reliable and includes Swedish norms (Kadesjo *et al.*, 2004).

Swanson Nolan and Pelham scale (SNAP-IV) (Swanson *et al.*, 2001) is a 26-item questionnaire for parents based on the DSM-IV criteria for ADHD (items 1–18), and oppositional defiant disorder (items 19–26). Each item can be scored on a four-graded Likert-scale; ‘Not at all’ = 0, ‘Just a little’ = 1, ‘Quite a bit’ = 2, and ‘Very much’ = 3. Scores are calculated for the three subscales ‘Inattentive’, ‘Hyperactive Impulsive’, and ‘Oppositional Defiant’ separately. Scores below 13 on the ‘Inattentive’ and ‘Hyperactive Impulsive’ scales, respectively, and scores below eight on the ‘Oppositional Defiant’ scale are considered normal. To equate scoring with ADHD criteria, scores of ‘0’ and ‘1’ can be classified as the absence of symptoms and ‘2’ and ‘3’ as the presence of symptoms (i.e. criterion is met). The psychometric properties have been considered acceptable.

### Physical/neurological examinations

The children were weighed and measured at 2.5 (T1) and 6 (T2) years of age, and head circumference was assessed at T1. The physical/neurological examination at T1 included kicking a ball and drawing a circle. At T2, the examination encompassed hand preference, ophthalmological status including ‘eye movements’, pupil reaction to light (direct and indirect), walking on tiptoes and heels, Fog’s test (walking on the lateral part of the feet), and jumping 10 jumps on each leg. To investigate deviations in fine and gross motor skills, coordination, speech, and social interaction at 2.5 and 6 years of age, guidelines regarding developmental milestones developed for paediatricians and general practitioners working at Swedish children’s health centres were used (rikshandboken-bhv.se, 2023). The first author (E.W.), a trained child psychiatrist who has been performing neurodevelopmental examinations for more than 30 years, performed all the physical/neurological examinations.

### Statistical analyses

Due to the small sample size non-parametric tests were performed. Correlations between gestational weeks and IQ were performed using Spearman’s correlation coefficient. Possible differences between boys and girls in terms of SDQ and ESSENCE-Q were calculated using the chi-square test and Fisher’s exact test due to the small number of cases. The related-samples Friedman’s two-way analysis of variance by ranks was used to compare means of the three Bayley subdomains and the means of the five WPPSI-IV indices. The SPSS package version 28 was used to conduct statistical analyses. *P*-values <0.05 were considered statistically significant. All significance tests were two-sided.

## Results

### Cognition

Bayley-III: At 2.5 years, the children performed within the normal range in all three Bayley subdomains (Table 2). The highest mean scores were observed in the Bayley Cognition subdomain. The means of the three subdomains were not statistically significantly different (*P* = 0.109).

**Table 2.** **Developmental assessment at age 2.5 years using Bayley test, third edition (Scales of Infant and Toddler Development)**.

Code number	Mothers code according to Brannstrom *et al.* (2022)	Sex	Age at first assessment (years)	Bayley Cognition (CI)	Bayley Language (CI)	Bayley Motor (CI)
1	1	M	2.5	115 (106–122)	115 (107–121)	100 (92–108)
2	3	M	2.6	90 (83–99)	71 (67–79)	91 (84–99)
3	2	M	2.6	115 (106–122)	100 (93–107)	100 (92–108)
4	4	M	2.7	125 (115–131)	106 (98–113)	103 (95–110)
5	5	F	2.6	125 (115–131)	115 (107–121)	110 (102–117)
6	3	F	2.7	115 (106–122)	124 (115–130)	124 (114–130)
7	6	F	2.4	125 (115–131)	121 (112–127)	100 (92–108)
8	5	M	2.8	135 (124–140)	106 (98–113)	110 (102–117)
9	NA	M	3.2	100 (92–108)	97 (90–104)	91 (84–99)
10	6	F	2.8	135 (124–140)	115 (107–121)	121 (112–127)
11	NA	M	2.6	105 (97–113)	112 (104–118)	115 (106–121)
Total mean (M:F)	NA	NA	2.7 (2.6:2.8)	116.8 (112.1:125.0)	107.5 (101.0:118.8)	105.9 (101.4:113.8)

The children with codes 2 and 6, 5 and 8, as well as 7 and 10 are siblings (not twins). The standard scores of the Bayley Cognition, Language and Motor all range from 40 to 160, with a mean score of 100 and a standard deviation of 15. −2 standard deviations or <70 are conventionally used as marker for moderate-severe delay. CI, confidence interval provides another means of expressing the precision of test scores; M, male; F, female; NA, not applicable due to not included in the publication by Brannstrom *et al.* (2022).

WPPSI-IV: At 6 years, the mean full-scale IQ was within the normal range (Table 3). The highest mean score on the WPPSI-IV test was the Fluid Reasoning Index. The largest confidence interval was in Verbal Comprehension index, suggesting the largest differences within the indices. The means of the five indices were not statistically significantly different (*P* = 0.252).

**Table 3.** Developmental assessment at age 6 years using the WPPSI-IV.

Code number	Mothers code according to Brannstrom *et al.* (2022)	Sex	Age at second assessment	WPPSI-IV Full Scale (CI)	Verbal Comprehension (CI)	Visuospatial (CI)	Fluid Reasoning (CI)	Working Memory (CI)	Processing Speed (CI)
1	1	M	6.2	93 (88–99)	87 (81–95)	100 (91–109)	86 (79–95)	90 (83–99)	93 (88–99)
2	3	M	6.2	119 (113–124)	132 (123–137)	97 (89–106)	119 (110–125)	108 (99–115)	90 (82–101)
3	2	M	6.5	99 (93–105)	96 (89–103)	115 (105–122)	113 (104–120)	90 (83–99)	102 (92–111)
4	4	M	6.6	111 (105–116)	99 (92–106)	106 (97–114)	104 (96–111)	111 (102–118)	105 (95–114)
5	5	F	6.3	107 (101–112)	93 (87–100)	106 (97–114)	113 (104–120)	99 (91–107)	111 (100–119)
6	3	F	6.2	113 (107–118)	123 (115–128)	121 (110–127)	122 (113–128)	93 (86–102)	87 (79–99)
7	6	F	6.2	98 (92–104)	90 (84–97)	112 (102–119)	116 (107–122)	90 (83–99)	105 (95–114)
8	5	M	6.2	100 (94–106)	84 (78–92)	94 (86–103)	122 (113–128)	111 (102–118)	93 (84–104)
Total mean (M:F)	NA	NA	6.3*	105 (97.6–112.4)	100.5 (85.9–115.1)	106.4 (98.6–114.1)	111.9 (101.9–121.9)	99 (91.0–107.0)	98.2 (91.1–105.4)

The children with codes 2 and 6, as well as 5 and 8 are siblings (not twins). WPPSI-IV, Wechsler Preschool and Primary Scale of Intelligence, 4th edition; the WPPSI-IV data include index score and confidence intervals. The mean score of WPSSI-IV Full scale and the five Primary Index Scales is 100; <70 is classified as ‘extremely low’ and represents <2% of the population. CI, confidence intervals; the interval indicates the degree of certainty of covering the true mean of the population; M, male; F, female.

* based on code numbers 1–6; Code numbers 9–11 have not been assessed at age 6 years.

### Parental questionnaires

SDQ: Supplementary Table S1 and Table 4 display the SDQ results at age 2.5 and 6 years, respectively. The mean total scores were normal at both ages. At age 2.5 years, two boys scored above the cut-off on the Emotional subscale, and one of them also scored above the cut-off on Peer problems, and on the total score. At age 6 years, the latter boy still scored above the cut-off regarding the Emotional subscale and the total score, and below the normal range on the Prosocial subscale, indicating social interaction problems. One boy scored above the cut-off regarding Hyperactivity both at age 2.5 years and age 6 years. Another boy had both Emotional and Peer problems at age 6 years, but none of these symptoms had been reported at age 2.5 years. Four boys and no girls had at one or two assessments, deviations regarding subscale or total scores. The difference between the sexes was not statistically significant (*P* = 0.213).

**Table 4.** Results from the questionnaires SDQ and ESSENCE-Q at age 6 years.

Code	Mothers code according to Brannstrom *et al.* (2022)	Age at second assessment	Sex	SDQ Total	SDQ Emotional	SDQ Conduct	SDQ Hyperactivity	SDQ Peer problems	SDQ Prosocial*	ESSENCE-Q^d^	ESSENCE-Q above cut-off^e^
1	1	6.2	M	5	**3**	0	0	**2**	10	1	
2	3	6.2	M	**11**	**6**	1	3	1	**5**	4^a^	X
3	2	6.5	M	9	0	2	**6**	1	10	0^b^	X
4	4	6.6	M	3	1	1	1	0	10	1	
5	5	6.3	F	2	1	0	1	0	8	0	
6	3	6.2	F	3	0	0	3	0	9	1	
7	6	6.2	F	1	1	0	0	0	9	0	
8	5	6.2	M	7	2	0	**4**	1	9	0^c^	
Mean (SD)	NA	6.3 (0.17)	NA	5.13 (3.56)	1.75 (1.98)	0.50 (0.76)	2.25 (2.12)	0.63 (0.74)	8.75 (1.67)	0.87 (1.36)	

The children with codes 2 and 6, as well as 5 and 8 are siblings (not twins). SDQ, Strengths and Difficulties Questionnaire; The range for the SDQ subscales are Emotional: 0–2, Conduct: 0–2, Hyperactivity: 0–3, Peer problems: 0–1, and Prosocial: 8–10; ESSENCE-Q: The early symptomatic syndromes eliciting neurodevelopmental clinical examinations-questionnaire; A cut-off score of ≥2 ‘yes’ or ≥3 ‘maybe’ has been suggested indicating ESSENCE pathology (Hatakenaka *et al.*, 2016); NA, not applicable.

* Note: SDQ Prosocial is not included in SDQ Total; numbers in bold indicate above the cut-off on the SDQ.

a 7 points if items where the parent was uncertain were included.

b 3 points if items where parent was uncertain were included.

c 1 point if item where parent was uncertain was included.

d Only including ‘yes’ responses.

e X indicates above the cut-off, either ≥2 ‘yes’ or ≥3 ‘maybe’.

ESSENCE-Q: Three boys scored above the cut-off at age 2.5 years. At age 6 years, one of the boys continued to score above the cut-off. One of the boys has not yet reached the age of 6 years. Another boy, scored above the cut-off only at age 6 years. At one or both assessments, four boys and no girls scored above the cut-off, but the difference was not statistically significant (*P* = 0.213).

M-CHAT: One child had a score of one, indicating ‘Low risk’ for ASD. No other deviations were found.

SNAP-IV: All children except one boy scored below the cut-off for all subscales and the total scale. The boy scored at the cut-off regarding the Oppositional Defiant subscale (Supplementary Table S2).

FTF: Only one boy screened positive regarding two domains, ‘social competence’, and ‘psychiatric problems’. As regards the other children, only minor problems, e.g. time perception, were reported (Supplementary Table S2).

### Physical, neurological, and psychiatric status at 2.5 and 6 years of age

Table 5 shows the physical, neurological, and psychiatric symptoms at 2.5 and 6 years of age. One boy, who has only been examined at age 2.5 years, had suspected cryptorchidism. Three children had infection-induced asthma at age 2.5 years, but the symptoms were not reported at age 6 years. Speech problems were observed in five children at age 2.5 years, but the problems had subsided at age 6 years. At age 2.5 years, hyperactive traits were present in one girl and one boy. Two boys had temper tantrums. At age 6 years, two boys had minor neurological signs. One boy, with previous hyperactive traits, was considered hyperactive at age 6 years, as confirmed by data from the SNAP-IV and SDQ. One boy exhibited vocal tics at age 6 years.

**Table 5.** Deviances in physical, neurological, and psychiatric status at 2.5 and 6 years of age.

Number	Sex	T1: Physical/neurological deviations	T1: Psychiatric deviations	T2: Physical/neurolo-gical deviations	T2: Psychiatric deviations
1	M	Inguinal hernia surgery at the age of 4 months	–	Positive Fog’s test*	–
2^a^	M	Hypotone oral motor skills	Speech delay Temper tantrums Poorer social skills	–	–
3	M	Asthma due to cold or flu RS-virus infection at age 1.5 years	Hyperactive tendency Unclear speech	–	Hyperactive
4	M	Tends to walk on his tiptoes Asthma due to cold or flu Left handed	Tends to have an unclear speech	Obstipation Left handed Positive Fog’s test*	–
5^b^	F		Tends to have an unclear speech	–	–
6^a^	F	–	–	–	–
7^c^	F	–	–	Unilateral tear duct surgery	–
8^b^	M	Left handed	–	Left handed	Child care staff had previously suspected speech delay, but the child had age adequate speech at examination. Vocal tics for at least 3 months
9	M	Suspected chryptorchidism; only one palpable testis in scrotum^d^	Temper tantrums	NA	NA
10^c^	F	–	Hyperactive tendency at the end of the examination	NA	NA
11	M	Asthma due to cold or flu Allergic to pine- and pistachio nuts (exanthema) History of fungal infections in arm- and knee fold and groin	Tends to have an unclear speech	NA	NA

T1: First examination at age 2.5 years; T2: second examination at age 6 years.

a Siblings but not twins.

b Siblings but not twins.

c Siblings but not twins.

d Based on information from examination at child health centre; NA: not applicable because the child had not reached the age of 6 years at the time the study ended.

* Fog’s test is a neurological examination where the child must walk on the lateral sides of the feet and arms/hands are expected to hang vertically along the body. If the child instead flexes the elbows and wrists, the finding is considered pathological and is labelled as ‘positive Fog’s test’.

### Comparison cognition and psychomotor development at 2.5 and 6 years

One child with the lowest result on Bayley-III Expressive Language (71 composite score, Table 2) had a catch-up regarding language at age 6 years, exhibiting the highest score of all children on Verbal Comprehension (132 index score, Table 3). This child had speech problems at age 2.5 years and had been assessed by a speech pathologist, but at age 6 years no speech problems were observed.

## Discussion

In the present study, we prospectively investigated the cognitive, developmental, physical, and psychiatric status of children aged 2.5 and 6 years born after UTx. The children performed cognitively within the normal range at both time points. Developmental and behavioural symptoms were seen in some children and affected boys exclusively. Asthma and speech problems were relatively common at age 2.5 years but subsided later.

The children performed cognitively within the normal range at both ages. At 2.5 years of age, parents of five children (four boys and one girl) reported that their child had poor expressive language, but according to the Bayley-III, Expressive language results were normal in all but one boy. This boy had a remarkable catch up at age 6 years. Cognitively, this child did not demonstrate any other developmental delays. Low results on Expressive language were also reported in a boy, in a case series study of two 3-year-old children born after UTx in Czech Republic (Janota *et al.*, 2023). As an isolated finding, it is common for boys under the age of 3 years to perform poorly in the Expressive language domain, and then to catch up (Krogh and Væver, 2019). Children born preterm are at greater risk of developmental and behavioural problems, especially those born extremely or very preterm (<32 weeks gestational age) (Pascal *et al.*, 2018) whereas children born at 32–36 gestational weeks are at moderately higher risk of neurodevelopmental impairments (Schonhaut *et al.*, 2015; Kong *et al.*, 2025). In the present study, the majority of the children were born late preterm or at term and showed no major cognitive deviances.

According to the SDQ, SNAP-IV, and ESSENCE-Q, the boys in the cohort tended to exhibit more neurodevelopmental deviances than the girls, but the differences were not statistically significant. In line with the present study, most neurodevelopmental disorders including ADHD, ASD, and tic disorders are in general overrepresented in males (Knight *et al.*, 2012; APA, 2013). One of the boys in the sample was considered hyperactive at age 6 years, but no complete ADHD investigation was performed and therefore we cannot tell whether diagnostic criteria for ADHD were met. Another boy exhibited vocal tics at age 6 years. According to a meta-analysis, chronic vocal tics in children have a prevalence of 0.69%, and transient tic disorders are more common (∼3%) (Knight *et al.*, 2012). In the present study, the tics of this boy had been present for at least 3 months but were only observed at age 6 years. Based on the current data, we cannot yet draw any conclusions about whether the tics are transient or chronic (APA, 2013). No other studies of children born after UTx have reported on tics. Typical onset of tics occurs after 3 years of age, and the present study is the first study of its kind to report status of UTx children at age 6 years.

Poor communicative skills were observed among 3 of 12 children aged 12 months (2 boys, 1 girl) in a study from the Dallas UTx centre. Nonetheless, at age 24 months, when five of the children were reexamined, all but one boy, demonstrated age-appropriate speech (Schulz *et al.*, 2022). In our sample, at age 2.5 years, speech problems were common. However, speech problems had subsided among those followed up at age 6 years. The fact that some children in our sample were born preterm, late preterm in most cases, does not appear to be the main cause of the language delay, as healthy preterm children usually exhibit normal speech development (Perez-Pereira, 2021).

The parental questionnaires revealed a tendency towards more behavioural problems among the boys compared to the girls. Three boys, but no girl, scored above the cut-off for Emotional problems on the SDQ at one or both of the assessment occasions. High scores on the Emotional problems subscale indicate symptoms of anxiety and depression. Boys do normally score higher than girls on the SDQ (Dahlberg *et al.*, 2020). Temper tantrums were also only affecting boys (N = 2). The study by Schulz *et al.*, which followed five children up to 24 months, did not report any behavioural problems and no differences between males and females (Schulz *et al.*, 2022). However, the Dallas group used information based on the Bright Futures Guidelines, a screening tool launched for childcare settings (Hagan *et al.*, 2017). The guidelines contain neither comprehensive cognitive assessments nor instruments for in-depth screening for general child psychopathology. Therefore, a tendency towards more behavioural problems among the males in their sample may have been missed due to both a less thorough examination and to a younger group of offspring.

Suspected cryptorchidism was reported in one boy at age 2.5 years. In the publication by Schulz *et al.* (2022), one boy was diagnosed with cryptorchidism. In a Swedish nationwide study, the cumulative prevalence of childhood cryptorchidism was 1.8%, with a higher prevalence in boys who were born preterm, small for gestational age (SGA) or with low birth weight (Bergbrant *et al.*, 2018). The boys in the present study could be considered a high-risk group for cryptorchidism since the majority were born preterm. However, none of the boys were SGA (Brannstrom *et al.*, 2022). The ARTs used in the present study also inherently carry an elevated risk of genitourinary malformations (Venetis *et al.*, 2023). In the study by Schulz *et al.*, a girl had an anteriorly/caudally displaced urethra. Urinary tract malformations, detected via ultrasound, have been observed in children born after a maternal kidney transplant, although the underlying maternal kidney disease may also contribute to the complications (Willis *et al.*, 2000). Schulz *et al.* (2022) performed postnatal ultrasound on all offspring; all females had a uterus and no renal malformations in the offspring were found. In the present study, the uteri of the female offspring had not yet been examined. No other malformations were observed in our case series (Brannstrom *et al.*, 2022).

Three boys had infection-induced asthma at age 2.5 years. Two of them participated in the 6-year examination and no longer had respiratory symptoms. Follow-up studies of the outcomes of offspring after maternal renal organ transplants have noted 4–10% of asthma cases in children (Willis *et al.*, 2000; Norrman *et al.*, 2015). In the general population in Sweden, 20–30% of 1- to 2-year-old children suffer from infection-induced asthma. Boys are affected more often, and the symptoms usually disappear between the ages of 3 and 7 years. The finding of three boys with infection-induced asthma at 2.5 years of age therefore appears to be an expected finding at that age, especially since no asthma was reported later. However, all children were born by Caesarean section, a procedure that carries a 1.3-fold increased risk of developing asthma (Thavagnanam *et al.*, 2008). Being born preterm, another risk factor for childhood asthma, may also have contributed to the asthma cases in our sample (Zhou and Tang, 2025).

### Strengths and weaknesses

This study has several strengths. First, the present study is a consequence of the pioneer research on UTx by Brannstrom *et al.* (2015), resulting in the first child born after UTx. Second, this study, a case series of 11 children, includes the world’s first live birth after UTx. Third, the number of participants is quite large; no other study within the same research area has examined so many children at age 2.5 years. No other study has so far prospectively followed the majority of their sample of children after UTx until the age of 6 years. The prospective design remains quite rare among studies reporting on offspring outcomes after maternal SOTs (Meinderts *et al.*, 2022). Fourth, we had no attrition; all children born after UTx in our case series who had reached the age of 2.5 and 6 years participated. Finally, the children were examined using validated and widely used instruments.

Weaknesses of the study include the relatively small sample size, and the lack of a comparison group consisting of children born after normal pregnancy and delivery by Caesarean section. Another suitable comparison group would be the offspring of mothers who have undergone IVF, without a history of UTx. The small sample does not offer enough statistical power, and no firm conclusions can therefore be drawn based on the reported deviances. However, in the future, each abnormality that has been observed can be of importance when data from multiple case series are pooled in a systematic review. No information on the children was collected from preschool/school regarding neurodevelopmental symptoms such as communicative skills, attention, and hyperactivity. A final weakness concerns the lack of follow-up data for the three children who were not assessed at age 6 years because data collection ended in September 2023, when these children had not yet reached age 6 years.

## Conclusions

For children born after UTx, and who were followed up at 2.5 and 6 years of age, the outcomes seemed generally favourable. Cognitive function was on par with their chronological age. However, some minor developmental and behavioural symptoms were seen by age 2.5 years and exclusively in the boys, but the symptoms had usually subsided by age 6 years. The results from this long-term follow-up study allow us to see with confidence how this unique group of children continues to develop, although the small sample size limits the ability to generalize the findings to all children born after UTx.

## Supplementary Material

deaf178_Supplementary_Table_S1

deaf178_Supplementary_Table_S2

## Data Availability

The data underlying this article cannot be shared publicly due to the privacy concerns for the individuals that participated in the study. The data will be shared on reasonable request to the corresponding author.
