# Supplementary material for: Uterus transplantation; first data on neurologic, neuropsychiatric, and physical examination follow-up of children up to 6 years of age
Source: Hum Reprod. 2025 Sep 13;40(12):2419–29. doi: 10.1093/humrep/deaf178 (PMC12675409; doi:10.1093/humrep/deaf178)
Supplement: deaf178_Supplementary_Table_S1 [file deaf178_supplementary_table_s1.pdf]

*Human Reproduction*, 2025, 1–1
https://doi.org/10.1093/humrep/deaf178
Supplementary Data

**Supplementary Table S1.** Results from the questionnaires SDQ and ESSENCE-Q at age 2.5 years.

| Code | Mothers' codes of laparotomy (1–6) and of robotic (#1–#2) UTx trials; (y, z) | Mothers code according to Brannstrom *et al.* (2022) | Age at first assessment | Sex | SDQ Total | SDQ Emotional | SDQ Conduct | SDQ Hyperactivity | SDQ Peer problems | SDQ Prosocial* | ESSENCE-Q[b] | ESSENCE-Q above cut-off[c] |
|---|---|---|---|---|---|---|---|---|---|---|---|---|
| 1 | 1[y] | 1 | 2.5 | M | 3 | 0 | 0 | 2 | 1 | 7 | 0 | |
| 2 | 3[y] | 3 | 2.6 | M | **12** | **3** | 3 | 2 | **4** | 7 | 2 | X |
| 3 | 2[y] | 2 | 2.6 | M | 9 | 0 | 3 | **5** | 1 | 9 | 0 | |
| 4 | 4[y] | 4 | 2.7 | M | 1 | 0 | 0 | 1 | 0 | 10 | 1 | |
| 5 | 5[y] | 5 | 2.6 | F | 2 | 0 | 0 | 2 | 0 | 9 | 0 | |
| 6 | 3[y] | 3 | 2.7 | F | 5 | 1 | 3 | 1 | 0 | 7 | 1 | |
| 7 | 6[y] | 6 | 2.4 | F | 2[a] | 1 | 1 | 0[a] | 0 | 9 | 1 | |
| 8 | 5[y] | 5 | 2.8 | M | 4 | **2** | 0 | 2 | 0 | 7 | 3 | X |
| 9 | #2[z] | NA | 3.2 | M | 4 | 0 | 2 | 1 | 1 | 7 | 0 | |
| 10 | 6[y] | 6 | 2.8 | F | 1 | 0 | 0 | 1 | 0 | 8 | 0 | |
| 11 | #1[z] | NA | 2.6 | M | 4 | 1 | 0 | 2 | 1 | 10 | 2 | X |
| Mean (SD) | | NA | 2.7 (0.20) | | 4.27 (3.4) | 0.73 (1.0) | 1.09 (1.4) | 1.73 (1.3) | 0.73 (1.2) | 8.18 (1.2) | 0.73 (1.0) | |

SDQ: Strengths and Difficulties Questionnaire; The Swedish norms for the SDQ for 3-year-olds vary between subscales; Emotional: 0–1; Conduct: 0–3; Hyperactivity: 0–3; Peer problems: 0–1; Prosocial: 7–10; ESSENCE-Q: The early symptomatic syndromes eliciting neurodevelopmental clinical examinations-questionnaire; A cut-off score of ≥2 'yes' or ≥3 'maybe' has been suggested indicating ESSENCE pathology (Hatakenaka *et al.*, 2016); NA: not applicable.
*Note: SDQ Prosocial is not included in SDQ Total; numbers in bold indicate above the cut-off on the SDQ.
[a] One item missing.
[b] Only including 'yes' responses.
[c] X indicates above the cut-off, either ≥2 'yes' or ≥3 'maybe'.
[y] Brannstrom *et al.* (2022).
[z] Brannstrom *et al.* (2020).
