# Supplementary material for: Uterus transplantation; first data on neurologic, neuropsychiatric, and physical examination follow-up of children up to 6 years of age
Source: Hum Reprod. 2025 Sep 13;40(12):2419–29. doi: 10.1093/humrep/deaf178 (PMC12675409; doi:10.1093/humrep/deaf178)
Supplement: deaf178_Supplementary_Table_S2 [file deaf178_supplementary_table_s2.pdf]

OXFORD

human reproduction

Human Reproduction, 2025, 1–1
https://doi.org/10.1093/humrep/deaf178
Supplementary Data

Supplementary Table S2. Results SNAP-IV and FTF questionnaires at age 6 years.

Code	Mothers code according to Brannstrom *et al.* (2022)	Sex	Age at second assessment	Total ADHD score	Inattentive score	Hyperactive Impulsive score	Oppositional Defiant score	FTF screen-positive
1	1	M	6.2	0	0	0	–	–
2	3	M	6.2	8	1	7	7	Domains: Social competence Psychiatric problems Subdomain: Obsessive behaviour
3	2	M	6.5	16	4	12	**8**	Subdomain: Time perception
4	4	M	6.6	3	1	0	2	–
5	5	F	6.3	11	3	5	–	–
6	3	F	6.2	6	3	2	1	–
7	6	F	6.2	7	4	1	2	Subdomain: Time perception
8	5	M	6.2	15	6	6	1	–
Mean (SD) (averaged by numbers of items)*	NA	NA	6.3 (0.17)	8.25 (5.55) (0.46)	2.75 (1.98) (0.31)	4.13 (4.19) (0.46)	3.50 (3.15) (0.44)	NA

SNAP, Swanson, Nolan and Pelham (SNAP) questionnaire (Swanson *et al.*, 2001); Scores below 13 on the 'Inattentive' and 'Hyperactive Impulsive' scales, respectively, and scores below eight on the 'Oppositional Defiant' scale are considered normal. Numbers in bold indicate above the cut-off on the SNAP-IV; FTF, Five-to-fifteen questionnaire; M, male; F, female.

* only applies to the SNAP-IV mean score; NA, not applicable.

© The Author(s) 2025. Published by Oxford University Press on behalf of European Society of Human Reproduction and Embryology.
This is an Open Access article distributed under the terms of the Creative Commons Attribution License (https://creativecommons.org/licenses/by/4.0/), which permits unrestricted reuse, distribution, and reproduction in any medium, provided the original work is properly cited.
Human Reproduction, 2025, 00, 1–11
https://doi.org/10.1093/humrep/deaf178
Original Article
